# Suicidal risk and resilience in juvenile fibromyalgia syndrome: a cross-sectional cohort study

**DOI:** 10.1186/s12969-020-00487-w

**Published:** 2021-01-06

**Authors:** Sabrina Gmuca, Maitry Sonagra, Rui Xiao, Kimberly S. Miller, Nina H. Thomas, Jami F. Young, Pamela F. Weiss, David D. Sherry, Jeffrey S. Gerber

**Affiliations:** 1 Department of Pediatrics, Division of Rheumatology, Children’s Hospital of Philadelphia, Philadelphia, USA; 2 Center for Pediatric Clinical Effectiveness, Children’s Hospital of Philadelphia, Philadelphia, USA; 3 PolicyLab, Children’s Hospital of Philadelphia, Philadelphia, USA; 4 University of Pennsylvania Perelman School of Medicine and Children’s Hospital of Philadelphia, Philadelphia, USA; 5 Children’s Hospital of Philadelphia, Roberts Center for Pediatric Research, 2716 South Street, 11214, Philadelphia, PA 19146 USA; 6 Department of Biostatistics, Epidemiology and Informatics, Perelman School of Medicine at University of Pennsylvania, Philadelphia, USA; 7 Center for Phenomic Science, Children’s Hospital of Philadelphia, Philadelphia, USA; 8 Department of Child and Adolescent Psychiatry and Behavioral Services, Children’s Hospital of Philadelphia, Philadelphia, USA; 9 Department of Pediatrics, Division of Infectious Diseases, Children’s Hospital of Philadelphia, Philadelphia, USA

**Keywords:** Resilience, Suicidality, Adolescence, Chronic pain, Juvenile fibromyalgia

## Abstract

**Background:**

To characterize suicidality among youth with juvenile fibromyalgia syndrome (JFMS) receiving treatment from pediatric rheumatologists at a tertiary care center in order to determine the prevalence of suicidality in JFMS and to explore risk factors for persistent suicidal ideation.

**Methods:**

We performed a cross-sectional cohort study of children 12–17 years old with JFMS seen in a specialty pediatric rheumatology pain clinic from 7/2017–9/2019. All subjects completed patient-reported outcomes measures, complemented by retrospective chart review. Subjects who endorsed item 8 on the Children’s Depression Inventory, 2nd Edition (CDI-2) were categorized as endorsing suicidal ideation. We assessed for differences between the suicidal and non-suicidal patients using Wilcoxon-rank sum test. Logistic regression modeling was performed to identify psychosocial factors associated with suicidality.

**Results:**

Of the 31 subjects, more than one-quarter endorsed suicidality. Nearly 90% of teens with suicidal ideation were established in outpatient counseling. In bivariate analyses, suicidality was associated with lower resilience and greater depression and anxiety (all *p* < 0.05). Pain intensity trended towards a statistically significant positive association (OR: 1.16 [0.99–1.37]; *p* = 0.06). Lower resilience was independently associated with suicidality (OR: 0.90 [95% CI: 0.82–0.98]; *p* < 0.02).

**Conclusions:**

Suicidality was prevalent among youth with JFMS and persistent despite concurrent receipt of mental health services. Higher patient-level resilience was independently associated with a reduced odds of suicidality. Future work should examine the role of resilience training on reducing psychological distress and mitigating the risk of suicidality in JFMS.

## Background

Our knowledge regarding the risk of suicidality in adult chronic non-cancer pain is well established. Specifically, significant data regarding suicidality in adults with fibromyalgia syndrome exist. In a retrospective cross-sectional study from Spain, suicidal ideation was endorsed in nearly 50% of adults with fibromyalgia syndrome [1]. Risk factors that have been identified as most strongly associated with suicidal ideation among adults with fibromyalgia syndrome include depression, anxiety, poor sleep quality, poor mental health and perceived burdensomeness of the disease [1–5]. One study found depression [2] and another found perceived burdensomeness to be independent predictors of suicidal ideation in this patient population [3]. In contrast, pain itself has not consistently been shown to be associated with suicidal ideation in adult fibromyalgia [1]. Therefore, suicidal ideation appears to be highly prevalent among severely affected adult fibromyalgia patients and more associated with mental, rather than physical manifestations [1]. Addressing suicidality is of the utmost importance due to the increased mortality by suicide reported in adults with fibromyalgia syndrome [6, 7].

Suicidal ideation, while reported to be prevalent among youth with chronic pain syndromes [8], has not, to our knowledge, been systemically evaluated in youth with juvenile fibromyalgia syndrome (JFMS) although it is one of the most common pediatric chronic non-inflammatory musculoskeletal pain syndromes. Affected youth exhibit significant physical disability and mental health problems in the setting of chronic widespread pain. Similar to adults, youth with fibromyalgia experience high rates of co-morbid mental health disorders including anxiety and depression [9]. Further exploration of predictors of suicidality can provide insight into ways to mitigate the potential risk for self-harm and mortality from suicide among affected youth.

Resilience serves as an important protective factor against psychological distress [10–12]. Resilience can be defined as a dynamic process of positive adaptation or continued development in the context of adversity (e.g. chronic pain) [11, 13]. Greater resilience is associated with improved physical function and reduced depression and pain in adult fibromyalgia syndrome [14]. Similarly, resilience is associated with fewer symptoms in youth with chronic musculoskeletal pain [15]. However, resilience has not specifically been assessed in youth with JFMS and its relationship with suicidal ideation remains unknown.

The aims of this study, therefore, were to characterize suicidality among youth with JFMS receiving treatment from pediatric rheumatologists at a tertiary care center in order to explore risk factors for persistent suicidal ideation. We hypothesized that resilience would be protective against suicidal ideation. Better understanding of not only risk but also protective factors for suicidal ideation could provide insight into novel treatment modalities (e.g. resilience training interventions) [16–20] to potentially reduce suicidality and associated mental health co-morbidities in JFMS.

## Patients and methods

### Study population

This was a cross-sectional cohort study of patients 12–17 years old diagnosed with JFMS according to the 2010 American College of Rheumatology criteria for fibromyalgia syndrome [21] and one of their parents/legal guardians, seen in either the general pediatric rheumatology clinic or pediatric rheumatology pain clinic at a tertiary care pediatric hospital from July 1, 2017 through October 1, 2019. The study received Institutional Review Board approval.

#### Inclusion criteria

Included patients received a primary or provisional diagnosis of JFMS (in accordance with the 2010 American College of Rheumatology Fibromyalgia Syndrome criteria [21]) by a physician at the time of their 1) initial consultation in either the general pediatric rheumatology clinic or the subspecialty pediatric rheumatology pain clinic, or 2) follow-up visit in the pediatric rheumatology pain clinic. Patients, and their proxies, were required to read or understand English well enough to complete study assessments, proxies had to provide permission (informed consent) for both self and child subjects, and children needed to assent or consent to participation.

#### Exclusion criteria

Subjects and/or proxies whose current medical status or cognitive functioning precluded completion of the assessment instruments (e.g., interfering acute/chronic medical conditions, blindness, hearing impairments, or prior concussion) as determined by the principal investigator were excluded. We also excluded subjects who had one or more active prescriptions for a stimulant medication so as not to confound or bias performance on the cognitive functioning measure (The PedsQL Cognitive Functioning Scale). We excluded subjects who did not have a legal guardian available to provide consent and/or complete study measures.

### Study procedures

#### Data collected as part of routine clinical care

Demographics and clinical data collected as part of routine clinical care were abstracted from the electronic medical record for all subjects. This included the following: physical exam, vital signs, previous laboratory or imaging results, past medical and psychological history, and documentation of current and past medications as well as the following patient-reported outcome measures: The Functional Disability Inventory (FDI) [22–24], the visual analog pain scale (VAS) with 100 being maximal pain [25], the widespread pain index (WPI) [21, 26, 27], and the symptom severity scale (SSS) score [21, 26, 27]. In order to meet criteria for fibromyalgia syndrome, a patient must have a WPI ≥ 7 and SSS ≥ 5 or WPI 3–6 and SSS ≥ 9 (in addition to having symptoms for at least 3 months and the lack of a disorder that would otherwise explain the pain) [21].

#### Study measures

At the time of the study visit both patients and their consented parents completed a series of questionnaires. These study measures are listed in Table 1.

**Table 1:** Study Measures

Domain	Measure	Details	Scoring
Anxiety	Multidimensional Anxiety Scale for Children, 2nd Edition (MASC-2) [28–30]	Assessment of anxiety symptoms in youth ages 8–19 years	T-scores ≥60 indicate increased likelihood of at least one anxiety disorder in the subject.
Cognitive Function	The PedsQL Cognitive Functioning Scale [31–33]	Subscale of the PedsQL Multidimensional Fatigue Scale. Assesses cognitive functioning.	The questions are answered on a Likert scale (0–100), with higher scores indicating better health related quality of life (fewer cognitive problems).
Depression	Children’s Depression Inventory, 2nd Edition (CDI-2) [34]	Assessment of depressive symptoms in children ages 7–17 years	T-scores ≥65 identify potentially clinically depressed individuals.
Executive Function	Behavior Rating Inventory of Executive Function, 2nd Edition (BRIEF-2) [35]	Assessment of children’s executive function. Captures children’s views of their own executive functions, or self-regulation, in their everyday environment.	T scores from 60 to 64 are considered mildly elevated, and T scores from 65 to 69 are considered potentially clinically elevated.
Fatigue	The PedsQL Multidimensional Fatigue Scale [33]	The scale assesses multidimensional fatigue through 3 dimensions: general, sleep/rest, and cognitive fatigue.	The 5-point Likert-type scale is transformed to a 0 to 100 scale. A higher score indicates less symptoms of fatigue.
HRQoL	The Patient-Reported Outcomes Measurement Information System (PROMIS) Pediatric Global Health (PGH-7) Measure [36, 37]	Summary assessment of a child’s health representing an individual’s overall assessment of their health, focusing on physical, mental and social health components.	Raw scores are converted to T-score values with a mean score of 50 (standard deviation of 10).
Resilience	14-Item Resilience Scale (RS-14) [38–41]	Assess personal resilience, focusing on domains of purpose, perseverance, self-reliance, equanimity and authenticity.	Scores range from 14 to 98 with a higher score indicating greater resilience.

*PedsQL* Pediatric Quality of Life, *HRQoL* Health-related quality of life

### Data analysis

Patient demographics and clinical characteristics were summarized by median and interquartile range (IQR) for continuous variables and frequency and percentage for categorical variables. Subjects with elevated scores (≥ 65) on The Children’s Depression Inventory, 2nd Edition (CDI-2) or endorsing item 8 (suicidal thoughts) on the CDI-2 triggered the study’s mental health (MH) safety check. These patients were assessed by a study team member for active suicidal ideation at the time of the study visit and this clinical assessment was documented in the patients’ medical record. Subjects who endorsed item 8 (1 = I think about ending my life, but I would never do it; or 2 = I want to end my life) were categorized as endorsing suicidal ideation.

Difference in demographics, clinical characteristics, and psychosocial factors between suicidal and non-suicidal patients were assessed using Wilcoxon Rank Sum test for continuous variables and Fisher’s exact test for categorical variables. Bivariate analysis was performed using simple logistic regression for all variables of interest to assess association with suicidal ideation. Subsequent multivariable logistic regression modeling was performed using backwards stepwise selection with a significance cut-off of 0.10. All data analyses were performed using SAS 9.4 (Copyright© 2002–2012 by SAS Institute Inc., Cary, NC, USA.). Two-sided *p* values less than 0.05 were considered statistically significant.

## Results

A total of 31 subjects were included in final analyses. Demographics and clinical characteristics are shown in Table 2. Patients were primarily female (87%), Caucasian (81%), and non-Hispanic (90%). Median age at study enrollment was 15 years (IQR: 14–16). Adolescents had a median pain duration of 12 months (IQR: 6–36) and patients were moderately disabled with a median FDI score of 23 (IQR: 15–31) [42]. Median pain visual analog score (VAS) was 59 (IQR: 32–68).

**Table 2:** Demographics and Clinical Characteristics among Adolescents with Juvenile Fibromyalgia Syndrome (*N* = 31)

*Demographics*	
Age [median (IQR)]	15 (14–16)
Sex, Female [n (%)]	27 (87%)
Race, Caucasians [n (%)]	25 (81%)
Ethnicity, Non-Hispanic [n (%)]	28 (90%)
*Clinical Characteristics [median (IQR)]*	
Pain Duration	12 (6–36)
Pain VAS (Visual Analog Scale)	59 (32–68)
Widespread Pain Index (WPI)	11 (9–13)
Symptom Severity Score (SSS)	8 (7–9)
Functional Disability Inventory (FDI) (0–60)	23 (15–31)

**Fig. 1:**
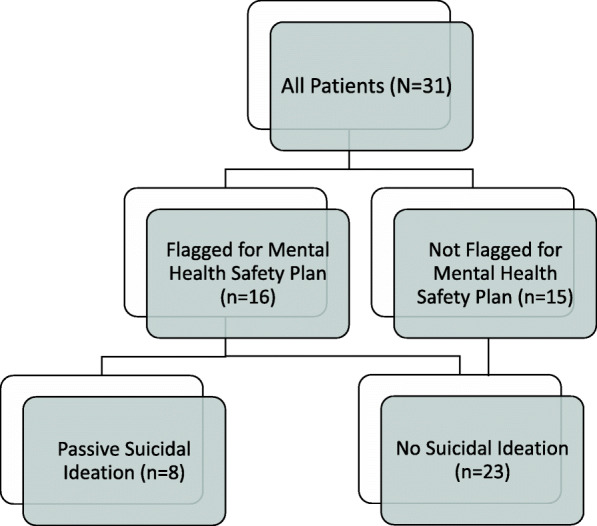
Population Flow Chart of Adolescents with Juvenile Fibromyalgia Syndrome Triggering Mental Health Safety Plan and Endorsing Suicidal Ideation. Legend. Flow diagram showing presence of suicidal ideation among those who did and did not flag positive on the mental health safety plan

Mental health co-morbidities were common among the cohort. Forty-five percent demonstrated concern for clinical depression (CDI-2 ≥ 65) and 65% for clinical anxiety (MASC-2 ≥ 60). Sixteen subjects (52%) triggered a mental health safety check (14 subjects had CDI-2 scores ≥65 of which 6 also endorsed item-8; and 2 endorsed item-8 only). A total of 8 subjects (26% of the entire cohort) endorsed suicidal ideation according to the CDI-2 but did not have an active plan or intent (Fig. 1).

**Fig. 2:**
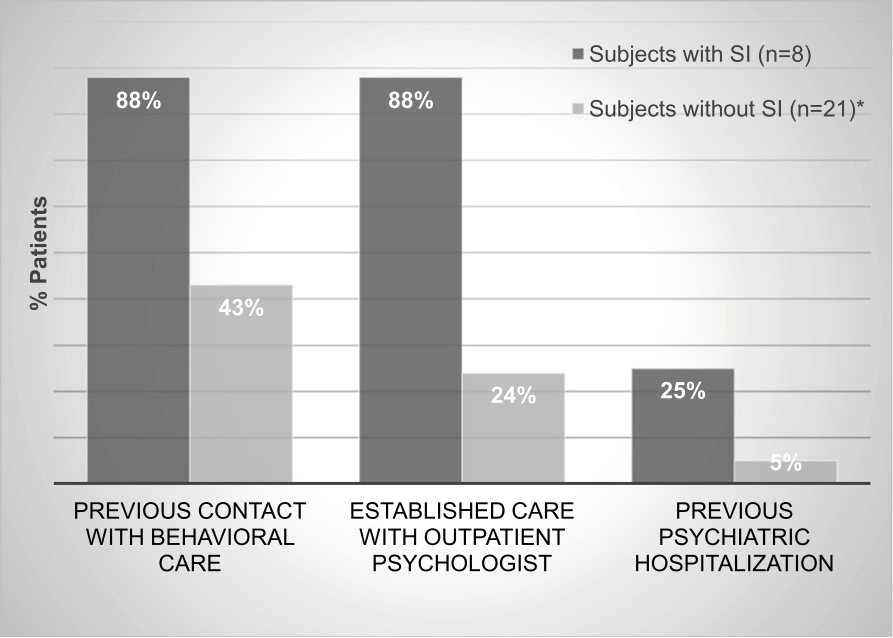
Mental Health Treatment Status in Patients Based on Suicidal Ideation among Adolescents with Fibromyalgia (*N* = 31). Legend. *Data on history of behavioral health status was missing for two subjects

Table 3 shows differences in clinical characteristics and study measures based on the presence of suicidal ideation. Adolescents with suicidal ideation reported significantly greater pain intensity with a median pain score of 64 (IQR: 60–75), compared to a median of 54 (IQR: 26–67) among those without suicidal ideation (*p* = 0.04). Regarding patient-reported outcomes measures (PROs), overall health-related quality of life (HRQoL), as measured by the PROMIS Global Health 7 (PGH-7) and functional disability (FDI), were similar between the patients with and without suicidal ideation (both *p* > 0.05). Symptoms of depression were greater among those with suicidal ideation with median scores on the CDI-2 of 74 (IQR: 63–83) vs. 55 (IQR: 51–67) among those with and without suicidal ideation, respectively (*p* = 0.02). Similarly, the suicidal ideation group had significantly greater symptoms of anxiety with a median score on the MASC-2 of 83 (IQR: 63–89) compared to a median score of 63 (IQR: 50–70) among those adolescents without suicidal ideation (*p* = 0.01). Resilience as measured by the RS-14 was significantly lower among those with suicidal ideation (median of 58 ([IQR: 43–69] vs. 74 [IQR: 67–81]; *p* = 0.02). Co-morbid somatic symptoms, pain duration, pain intensity, and measures of fatigue and cognitive functioning were all similar between the two groups (*p* > 0.05). Outpatient psychological counseling at the time of study participation was reported in 88% of those with suicidal ideation, and 25% (2 of 8) reported a previous psychiatric hospitalization (Fig. 2).

**Table 3:** Clinical Characteristics and Patient Reported Outcome Measures Based on The Presence of Suicidal Ideation Among Adolescents with Juvenile Fibromyalgia Syndrome (*N* = 31)

Variables	SI (*n* = 8)	No SI (*n* = 23)	*p*-value
*Clinical Characteristics [median (IQR)]*			
Pain Duration (months)	10 (5–24)	24 (10–36)	0.22
Pain VAS (Visual Analog Scale)	64 (60–75)	54 (26–67)	0.04^*^
Widespread Pain Index (WPI)	12 (9–13)	11 (9–14)	0.80
Symptom Severity Score (SSS)	8 (7–10)	8 (7–9)	1.00
*Patient Reported Outcome Measures (PROs) [median (IQR)]*			
Functional Disability Inventory (FDI) (0–60)	25 (16.5–31)	20 (15–31)	0.46
PROMIS Global Health 7 (PGH-7)	34 (28–40)	39 (34–42)	0.13
14-item Resilience Scale (14–98)	58 (43–69)	74 (67–81)	0.02^*^
CDI-2 (Depression)	74 (63–83)	55 (51–67)	0.02^*^
MASC-2 (Anxiety)	83 (63–89)	63 (50–70)	0.01^*^
PedsQL Total Multidimensional Fatigue Scale (MFS) (0–100)	37 (32–43)	39 (28–56)	0.80
MFS General Fatigue	29 (21–38)	42 (25–54)	0.21
MFS Sleep	31 (25–52)	42 (29–54)	0.39
MFS Cognitive	42 (40–63)	38 (25–71)	0.59
BRIEF-2 Global Executive Composite (GEC) T Score	57 (51–69)	58 (49–71)	1.00

Legend. *<0.05 = Significant *p*-value. *IQR* = interquartile range. Differences in characteristics were assessed using Wilcoxon Rank Sum test for continuous variables and Fisher’s Exact test for categorical variables. Pain VAS (visual analog scale) range from 0 to 100 with higher scores indicating more pain. SS scores total between 0 and 12 with higher scores indicating greater severity. WPI score ranges from 0 to 19 with higher values indicating greater involvement of different anatomical regions where the child has experienced pain over the past 7 days. Greater FDI scores indicate more functional disability. 14-item Resilience Scale - greater scores indicate greater resilience (ranging from 14 to 98). The Children’s Depression Inventory, 2nd Edition (CDI-2) is an assessment of depressive symptoms in children and adolescents ages 7–17 years, where T-scores ≥65 identify potentially clinically depressed individuals. The Multidimensional Anxiety Scale for Children, 2nd Edition (MASC-2) is a standardized, 50-item questionnaire assessing anxiety symptoms in adolescents where T-scores ≥60 indicate increased likelihood of at least one anxiety disorder in the subject. The Pediatric Quality of Life Inventory Multidimensional Fatigue Scale (PedsQL MFS) assesses 3 dimensions: general, sleep/rest, and cognitive fatigue. Scores ranges from 0 to 100 where higher scores indicate less symptoms/problems in a dimension. Behavior Rating Inventory of Executive Function-2 (BRIEF-2) is a standardized rating scale used to assess children’s executive functions in home and school environments where T scores from 60 to 64 are considered mildly elevated, and T scores from 65 to 69 are considered potentially clinically elevated

Table 4 shows both unadjusted and adjusted odds ratios (ORs) for the presence of suicidal ideation in the cohort. In bivariate analyses, depression (OR: 1.11 [95% CI: 1.10–2.10]) and anxiety (OR: 1.09 [95% CI: 1.01–1.17]) were positively associated with suicidal ideation and resilience was negatively associated with suicidal ideation (all *p* < 0.02). In adjusted analyses, resilience was independently associated with a decreased odds of suicidal ideation (OR: 0.90 [95% CI: 0.82–0.98]; *p* < 0.02) and pain intensity trended towards an increased odds of suicidal ideation (OR: 1.16 [0.99–1.37]; *p* = 0.06).

**Table 4:** Logistic regression model assessing variables associated with Suicidal Ideation among Adolescent with Juvenile Fibromyalgia Syndrome

Variables	Unadjusted ORs (95% CI)	*p*-value	Adjusted ORs (95% CI)	*p*-value
Pain Intensity (Pain VAS)	1.08 (1.00–1.17)	0.06	1.16 (0.99–1.37)	0.06
RS-14 (Resilience)	0.93 (0.87–0.99)	0.02^*^	0.90 (0.82–0.98)	0.02^*^
CDI-2 (Depression)	1.11 (1.01–1.20)	0.02^*^	–	–
MASC-2 (Anxiety)	1.09 (1.01–1.17)	0.02^*^	–	–

Legend. ^*^Significant *p*-value. *VAS =* visual analog scale, *RS-14* = 14-item Resilience Scale, ranging from 0 to 98, *CDI-2* = Children’s Depression Inventory, 2nd Edition, *MASC-2* = Multidimensional Anxiety Scale for Children, 2nd Edition. Depression was removed from the multivariable model due to multicollinearity. Backward selection method was used with stay selection level *p* < 0.1

## Discussion

More than one-quarter of adolescents with JFMS seen by pediatric rheumatologists endorsed suicidal ideation. Of those teens with suicidal ideation, nearly 90% had established outpatient counseling when suicidal ideation was expressed. Suicidal ideation was significantly associated with lower resilience and greater depression and anxiety. Pain intensity trended towards a statistically significant positive association with suicidal ideation. In adjusted analyses, resilience remained an independent negative predictor of suicidal ideation. With each one point increase on the RS-14, adolescents had a 10% lower odds of endorsing suicidal ideation.

The RS-14 is a continuous, quantitative measure of resilience that has established cutoffs for levels of resilience: very low (14–56), low (57–64), on the low end (65–73), moderate (74–81), moderately high (82–90), and high (91–98) [43]. We not only found statistically significant differences in levels of resilience based on the presence of suicidal ideation but we also found that youth with suicidal ideation fell into the low resilience category whereas youth with JFMS without suicidal ideation were categorized as having moderate resilience. Therefore, we have reason to believe that even modest increases in resilience can have potential mitigating effects on the risk of suicidality in JFMS. Since resilience is both measurable [44, 45] and mutable, routine use of a resilience measure such as the RS-14 in routine clinical care for JFMS might provide additional data to trigger a heightened awareness for risk for suicide. This might be beneficial given that youth with suicidal ideation might not overtly share their suicidal thoughts and tendencies with providers. Additionally, these findings suggest that the application of resilience training programs or resilience coaching might be of benefit for JFMS. Resilience training programs including individual- or group-based interventions, online-based modules, and multi-modal training have been proven to be effective in reducing psychological distress in adults with fibromyalgia syndrome as well as adolescents and young adults with chronic illness [12, 20, 46]. Furthermore, resilience training interventions are typically preventative and the fact that patients in our study reported suicidal ideation despite being established in mental health services underscores the need for earlier, preventative measures before suicidal ideation manifests.

The significant proportion of youth with JFMS endorsing suicidal ideation in this study is alarming, especially with the knowledge that a number of pharmacologic treatments for chronic pain can lead to the adverse side effect of suicidal ideation [47]. While we are limited in not having prescription medication data for this study, our division’s multidisciplinary treatment approach is non-pharmacologic and therefore we strongly suspect that reported suicidal ideation in our study was not associated with medication usage [47, 48]. Rather, our findings support refraining from the use of opioids in the treatment of JFMS as this can potentiate the underlying, existing risk of suicidal ideation in children and adolescents with JFMS.

Similar to adult studies, we did not find that pain or pain-related disability was related to suicidal ideation in adolescents with JFMS. We found that mental health burden and resilience, (similar to perceived burdensomeness), were the factors most strongly associated with suicidality. However, unlike the adult population, parental factors likely additionally contributed to a child’s mental health status and risk of suicidality. Future examination of parental resilience, parental psychological flexibility, and pain catastrophizing and their relationship to suicidality in JFMS would be helpful in further identifying potentially modifiable risk factors for suicidal ideation in JFMS.

This study has limitations. While resilience was found to be protective against suicidal ideation, we cannot draw conclusions regarding causality given the cross-sectional nature of the study. Furthermore, the prevalence of suicidal ideation in this cohort is a point prevalence rather than a lifetime prevalence. Longitudinal assessment of suicidal ideation in youth with JFMS would help determine the risk of suicidal ideation and mortality from suicide over one’s lifetime. Our modest sample size might have contributed to the non-significance of some factors that were examined and therefore we may not have detected all potentially clinically relevant risk factors for suicidality in JFMS.

## Conclusions

Suicidality is prevalent among youth with JFMS and persistent despite concurrent receipt of mental health services. Higher patient-level resilience was independently associated with a reduced odds of suicidality whereas pain and pain-related disability were not found to be risk factors for suicidal ideation. These findings underscore similarities regarding suicidality in juvenile and adult fibromyalgia syndrome. Future work should examine the role of resilience coaching on reducing psychological distress and mitigating the risk of suicidality in JFMS.

## Data Availability

The datasets used and/or analysed during the current study are available from the corresponding author on reasonable request.

## Abbreviations

BRIEF-2: Behavior Rating Inventory of Executive Function, 2nd Edition
CDI-2: Children’s Depression Inventory, 2nd Edition
FDI: Functional disability inventory
HRQOL: Health-related quality of life
IQR: Interquartile range
JFMS: Juvenile fibromyalgia syndrome
MASC-2: Multidimensional Anxiety Scale for Children, 2nd Edition
MH: Mental health
OR: Odds ratio
PedsQL: Pediatric quality of life
PROMIS PGH-7: Patient-Reported Outcomes Measurement Information System Pediatric Global Health
PROs: Patient-reported outcomes
RS-14: Resilience Scale 14-item
SSS: Symptom severity scale
VAS: Visual analog scale
WPI: Widespread pain index
